# The DEAD-Box RNA Helicase DDX3 Interacts with m^6^A RNA Demethylase ALKBH5

**DOI:** 10.1155/2017/8596135

**Published:** 2017-11-23

**Authors:** Abdullah Shah, Farooq Rashid, Hassaan Mehboob Awan, Shanshan Hu, Xiaolin Wang, Liang Chen, Ge Shan

**Affiliations:** ^1^CAS Key Laboratory of Innate Immunity and Chronic Disease, CAS Center for Excellence in Molecular Cell Science, School of Life Sciences, University of Science and Technology of China, Hefei, China; ^2^Anhui Province Key Laboratory of Brain Function and Brain Disease, Hefei, Anhui 230001, China; ^3^Department of Neurosurgery, Anhui Provincial Hospital Affiliated to Anhui Medical University, Hefei, Anhui 230001, China

## Abstract

DDX3 is a member of the family of DEAD-box RNA helicases. DDX3 is a multifaceted helicase and plays essential roles in key biological processes such as cell cycle, stress response, apoptosis, and RNA metabolism. In this study, we found that DDX3 interacted with ALKBH5, an m^6^A RNA demethylase. The ATP domain of DDX3 and DSBH domain of ALKBH5 were indispensable to their interaction with each other. Furthermore, DDX3 could modulate the demethylation of mRNAs. We also showed that DDX3 regulated the methylation status of microRNAs and there was an interaction between DDX3 and AGO2. The dynamics of m^6^A RNA modification is still a field demanding further investigation, and here, we add a link by showing that RNA demethylation can be regulated by proteins such as DDX3.

## 1. Introduction

DEAD- (Asp-Glu-Ala-Asp-) box (DDX) proteins are the largest family of RNA helicases [1]. This family plays pleiotropic functions in cells by an interaction with other proteins or different forms of RNA, to maintain the integrity of the secondary and tertiary structure of RNAs and facilitate multiple RNA processing procedures [1–3]. These helicases contain a highly conserved catalytic core domain that mediates the ATPase and helicase activities as well as the less conserved N and C-termini, which are thought to confer functional specificity and subcellular localization of individual DDX helicases [4–6]. DDX3 is multifunctional and is ubiquitously expressed in a wide range of tissues [7, 8]. The protein shuttles between the nucleus and cytoplasm and can localize to P-bodies under stress conditions [9–12]. Over the last years, DDX3 has been reported to play important roles in key biological processes like cell cycle progression, apoptosis, cancer, stress response, hypoxia, and response to radiation [8, 13–15]. DDX3 participates in many steps of RNA metabolism including RNA transcription, RNA splicing, mRNA transportation, and translation initiation [10, 16–19]. Multiple lines of evidence suggest that specific cofactors can modify the functionality of DDX3, such as DDX3 forms functional complex with the transcription factor SP1 (specificity protein 1) and enhances the expression activity of its cognate promoters [16].

The 6-methyladenosine (m^6^A) RNA methylation is the most prevalent and enriched modification of both coding and noncoding RNAs [20–26]. m^6^A accounts for about 50% of total methylated ribonucleotides and is present in 0.1%–0.4% of all adenosines in total cellular RNAs [20, 23, 24]. The presence of m^6^A affects nuclear retention, pre-mRNA splicing, stability of mRNAs, and stability of small RNAs [22, 27–29]. The knockdown of ALKBH5 enhances mRNA export to the cytoplasm [29], whereas the depletion of METTL3 inhibits mRNA export [30]. The demethylase FTO (fat mass and obesity associated) modulates alternative splicing by removing m^6^A around splicing sites and by inhibiting the binding of serine- and arginine-rich splicing factor 2 (SRSF2) [31]. The presence of m^6^A at the 5′ UTR (untranslated region) improves cap-independent translation [32], and eIF3 (eukaryotic initiation factor 3) is proposed to interact with m^6^A mark and facilitates ribosome loading [33].

m^6^A in mRNAs affects cell differentiation and the expression of numerous genes including transcription factors [34]. For instance, m^6^A affects the differentiation of preadipocytes during adipogenesis [31, 35]. Exposure of the breast cancer stem cell (BCSC) to hypoxia induces m^6^A demethylation of a key pluripotency factor, NANOG, by ALKBH5 [36]. Demethylation of NANOG increases transcript stability and promotes BCSC proliferation [36]. The depletion of methyltransferases *Mettl3* (methyltransferase-like 3) and *Mettl14* (methyltransferase-like 14) leads to low levels of m^6^A and reduced self-renewal of mouse ES cells [37].

The m^6^A modification is posttranscriptionally installed by a multicomponent methyltransferase complex with at least three core proteins, namely, METTL3, METTL14, and Wilms' tumor 1-associating protein (WTAP) [38, 39]. In recent years, the discoveries of the two RNA m^6^A demethylases, FTO and alkylated DNA repair protein AlkB homolog 5 (ALKBH5), demonstrate that the m^6^A RNA modification can be erased [29, 40].

In this study, we have showed that DDX3 and ALKBH5 interacted with each other and DDX3 could modulate the m^6^A demethylation of RNAs. Since the dynamics of m^6^A RNA modification was largely elusive, here, we demonstrated that RNA demethylation could be subjected to regulation.

## 2. Materials and Methods

### 2.1. Cell Culture and Transfection

HEK293T and HeLa cells were cultured in Dulbecco's Modified Eagle's Medium (DMEM) supplemented with 1% antibiotics and 10% fetal bovine serum (FBS). Cells were cultured at 37°C in a humidified 5% (vol/vol) CO_2_ incubator. All plasmid or siRNA transfections were carried out using Lipofectamine 2000 (Invitrogen) according to the manufacturer's protocol.

### 2.2. Plasmid Construction

All plasmids were constructed with recombinant method and/or restriction enzyme digestion and ligation. The full-length and all deletion constructs for DDX3 were cloned into p3xFLAG-Myc-CMV vector (Sigma-Aldrich). The full length and all deletion constructs for methyltransferases and demethylases were cloned into pKH3-HA vector. All constructs were confirmed by sequencing. Primer sequences used along with other oligo information were included in Supplementary Table S1 available online at https://doi.org/10.1155/2017/8596135.

### 2.3. Immunoprecipitation

The cells were washed twice with ice-cold PBS and cross linked with UV 12000 J/cm^2^ for 2 min in PBS. The cells were incubated on ice in modified RIPA lysis buffer (150 mM NaCl, 50 mM Tris pH 8.0, 1% Nonidet P-40, 0.5% deoxycholate, and a protease inhibitor mixture (Roche Applied Science, Mannheim, Germany)) for 20 min and rotated at 4°C for 10 min. Then, lysate was sonicated typically for 5 min at 30% amplitude, 3 sec pulses followed by 6 sec rest period. The lysates were clarified by centrifugation for 20 min at 14,000 rpm at 4°C. Fifty (50 *μ*l) of Dynabeads Protein G magnetic particles (Invitrogen) were resuspended, 500 *μ*l of lysis buffer. Then, 1.5 *μ*g of respective antibodies was added to the beads and incubated on a rotating wheel at room temperature for 30 min. Beads were precipitated by magnet and finally resuspended again in cleared cell lysates and incubated for 2 h at 4°C. In the last step, the beads were resuspended in 50 *μ*l of lysis buffer and subjected to Western blots.

### 2.4. Western Blotting

The proteins either from cell lyses or isolated from the IP beads were subjected to SDS-PAGE and then transferred on to a polyvinylidene difluoride (PVDF) membrane. Following blocking the membrane was probed using the corresponding primary antibodies overnight at 4°C. HA-Tag (C29F4) rabbit monoclonal, monoclonal anti-FLAG M2 (Sigma-Aldrich), mouse DDX3 (C-4) mAb, rabbit polyclonal anti-ALKBH5 (Sigma-Aldrich), and mouse anti-GAPDH antibody (Signalway) were used. After washing, the membrane was incubated with secondary horseradish peroxidase-conjugated goat anti-rabbit IgG (1 : 5000; ZB-2301) or anti-mouse IgG antibodies for 2 h at room temperature. Enhanced chemiluminescence substrates (EMD Millipore) were then applied, and signals were detected using a chemiluminescence imaging system (ChemiDoc™ MP Imaging System; Bio-Rad Laboratories Inc.).

### 2.5. RNA Extraction and Real-Time Quantitative PCR (qRT-PCR)

Total RNA from cells was isolated using TRIzol reagent (Invitrogen) according to the manufacturer's protocol. The RNA concentration was determined by spectrophotometer. cDNA for qRT-PCR was synthesized from total RNA by the GoScript Reverse Transcription System (Promega) according to the supplied protocol using random hexamer primer and oligo dT. qPCR was performed with the GO Taq qPCR Master Mix (Promega) on the PikoReal Real-time PCR System (Thermo Scientific) according to the standard procedures.

### 2.6. MicroRNA and Poly(A) mRNA Isolation

MicroRNA and poly(A) mRNA from cells were extracted using the mirVana™ miRNA Isolation Kit and PolyAT tract mRNA Isolation System (Promega), respectively, according to the manufacturer's protocol.

### 2.7. Protein Complex Modeling

The complex modelling was performed by docking ALKBH5_190–293_ to DDX3_211–402_ on a Rosetta web server (http://rosie.rosettacommons.org/docking2). The parameters used for docking are set to default [41–43]. Docking results are open to public. The docking job of ALKBH5_190–293_ to DDX3_211–402_ was assigned an ID (33161) or can be accessed with the following link http://rosie.rosettacommons.org/docking2/viewjob/33161. Model with the best score was analyzed with PyMOL software (The PyMOL Molecular Graphics System, Version 1.8 Schrödinger, LLC). Structures used for modelling are obtained from protein data bank, accession numbers are 2I4I for DDX3_211–402_ [44] and 4061 for ALKBH5_190–293_ [45].

### 2.8. Analysis of m^6^A Level Using Dot-Blot Assay

The m^6^A dot-blot was performed on a Bio-Dot Apparatus (Bio-Rad Laboratories Inc.). In brief, the RNA samples were denatured and spotted to nitrocellulose membrane under vacuum. After UV cross-linking, the membranes were baked at 80°C for 1 hr, and methylene blue staining was used to examine equal RNA loading. For detecting m^6^A levels, rabbit anti-m^6^A antibody (Millipore Sigma) was diluted with 1 : 500 in 0.1% TBST and 5% nonfat dry milk and incubated with the membranes overnight (4°C). Following extensive washing with 0.1% TBST, the blot was incubated with horseradish peroxidase- (HRP-) conjugated anti-rabbit IgG secondary antibody for 2 h at 25°C. The membrane was washed again with 0.1% TBST and visualized by ECL Western Blotting Detection Kit (Thermo Scientific). Dots were quantified with imageJ.

### 2.9. Cell Proliferation Assay

Cell viability was measured with the MTT Cell Proliferation and Cytotoxicity Detection Kit (Keygentec, Nanjing, China) according to the manufacturer's recommendations. Cells in 96-well plates were plated at a density of 2 × 10^3^ per well. Cells were then transfected with siRNAs or scrambled control. MTT reagents were added at indicated time points. Four hours later, the supernatant was removed, and DMSO was added to dissolve the blue precipitates. The number of live cells was determined by the OD value, which was measured by a plate reader (MultiSkan GO, Thermo Scientific).

## 3. Results

### 3.1. DDX3 Interacted with ALKBH5

**Figure 1:**
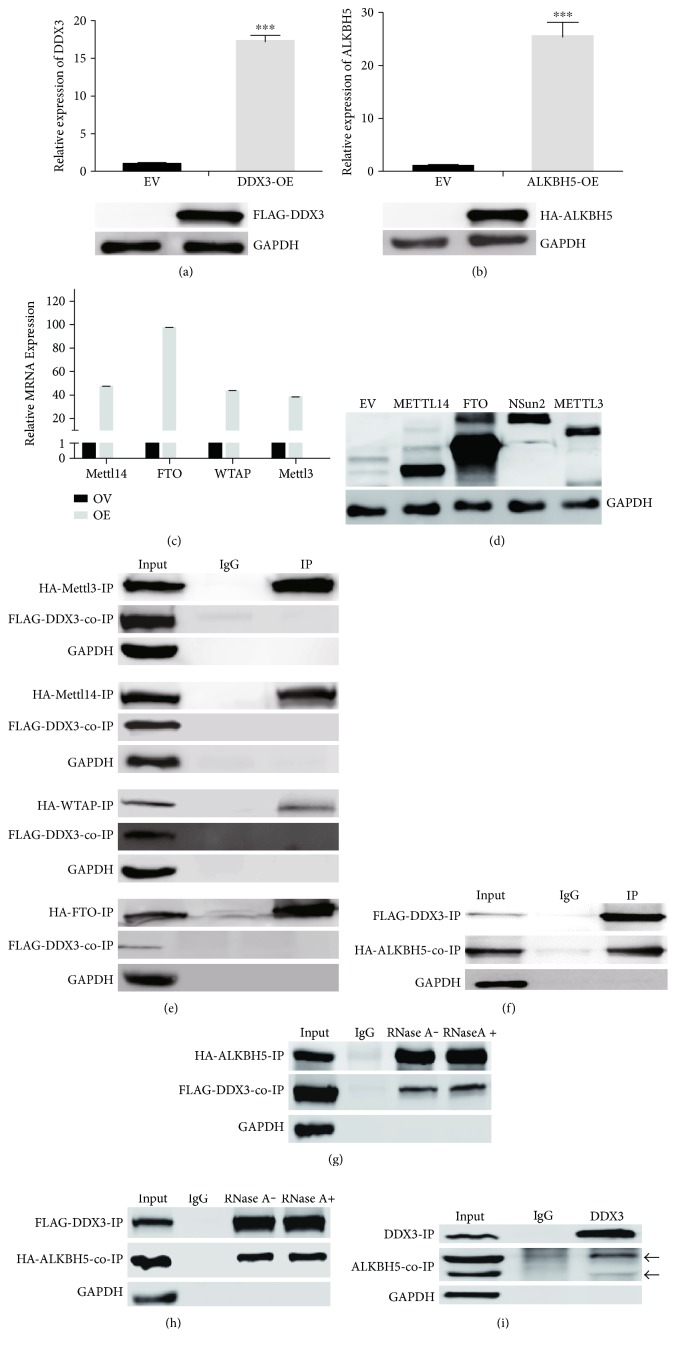
Identification of interaction between DDX3 and ALKBH5. (a) FLAG-DDX3 overexpression in HEK293T cells, relative DDX3 mRNA (upper panel), and protein levels (lower panel) are shown. (b) HA-ALKBH5 overexpression in HEK293T cells, ALKBH5 mRNA (upper panel), and protein levels (lower panel) are shown. (c) Overexpression of methyltransferases and demethylases in HEK293T cells, mRNA levels examined by qPCR. (d) Overexpression of the corresponding protein (indicated with arrowhead) examined by Western blots. (a–d) EV, empty vector; OE, overexpression. (e) DDX3 showed no interaction with the METTL3, METTL14, WTAP, or FTO, examined with IP and co-IP. (f) The interaction of ALKBH5 with DDX3 examined with FLAG-DDX3 IP and HA-ALKBH5 co-IP in HeLa cells. (g) The interaction of ALKBH5 with DDX3 in the presence or absence of RNase A, examined with HA-ALKBH5 IP and FLAG-DDX3 co-IP in HEK293T cells. (h) The interaction of ALKBH5 with DDX3 in the presence or absence of RNase A, examined with FLAG-DDX3 IP and HA-ALKBH5 co-IP in HEK293T cells. (i) Endogenous DDX3 interacted with endogenous ALKBH5, examined with IP and co-IP; two known isoforms of ALKBH5 are indicated with arrowheads. IP and co-IP were performed in triplicates, and representative results are shown. ^∗∗∗^*P* < 0.001; *P* values were determined with two-tailed Student's *t*-test; error bars represent standard deviation (SD).

To unveil the role of DDX3 in RNA methylation, we started to determine whether DDX3 physically interacted with m^6^A methyltransferases and demethylases. We constructed expression plasmids of METTL3, METTL14, WTAP, FTO, and ALKBH5. We cotransfected HEK293T cells with individual expression plasmids together with DDX3 expression plasmid (Figures 1(a), 1(b), 1(c), and 1(d)). The successful overexpression was confirmed by real-time qPCR and Western blot (Figures 1(a), 1(b), 1(c), and 1(d)). We then performed immunoprecipitation (IP) to examine their interaction with DDX3 (Figures 1(e), 1(f), 1(g), 1(h), and 1(i)). It was found that IP of METTL3, METTL14, WTAP, or FTO could not co-IP DDX3 (Figure 1(e)). In contrast, IP of ALKBH5 could co-IP DDX3 (Figures 1(f) and 1(g)). Furthermore, IP of DDX3 could co-IP ALKBH5 (Figure 1(f)). These data demonstrated that ALKBH5 was the only protein among the five methyltransferases and demethylases that interacted with DDX3.

The interaction between DDX3 and ALKBH5 was not RNA dependent, as the interaction was essentially unchanged with or without RNase A treatment (Figures 1(f) and 1(g)). We further examined whether they interacted with each other endogenously by performing IP with antibodies against DDX3 and indeed ALKBH5 could be co-IPed (Figure 1(h)).

### 3.2. ATP Domain of DDX3 Interacted with DSBH Domain of ALKBH5

**Figure 2:**
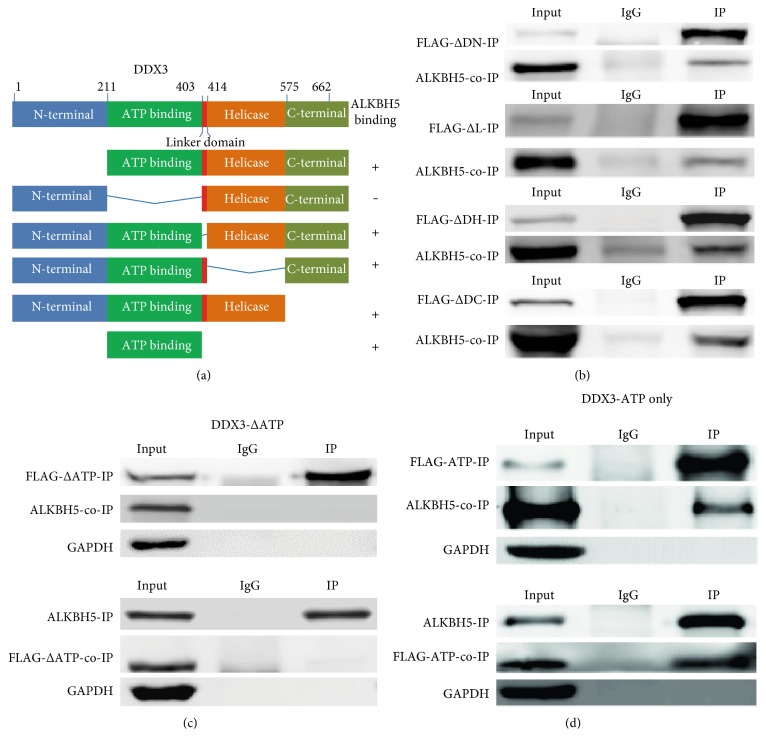
Determination of the binding domain of DDX3 with ALKBH5. (a) Schematic diagram of full-length DDX3 and the corresponding partial deletion constructs; summary of the results of interaction with ALKBH5 was also shown. (b) Deletion of N-terminal domain, Linker domain, Helicase domain, or the C-terminal domain of DDX3 showed interaction with the full-length ALKBH5, as examined with IP and co-IP. (c) The interaction was abolished when ATP domain of DDX3 was deleted, as examined with IP and co-IP. (d) ATP domain of DDX3 alone interacted with full-length ALKBH5, examined with IP and co-IP. All IP and co-IP were performed in triplicates, and representative results were shown.

We further asked which domain of DDX3 interacted with ALKBH5. For this purpose, 5 partially deleted constructs of DDX3 were constructed (Figure 2(a)). Each of these constructs was then used to perform IP, and full length ALKBH5 was then examined for the co-IP in HEK293T cells (Figures 2(a), 2(b), 2(c), and 2(d)). Among these constructs, deletion of either N-terminal domain, Linker domain, Helicase domain, or the C-terminal domain still showed interaction with the full length ALKBH5 (Figure 2(b)). However, upon deletion of the ATP-binding domain (DDX3-ΔATP), the binding with ALKBH5 was abolished (Figure 2(c)). IP of full length ALKBH5 also did not co-IPed DDX3-ΔATP (Figure 2(c)). Interestingly, the ATP-binding domain of DDX3 itself could interact with the full length ALKBH5 (Figure 2(d)). These results showed that ATP--binding domain but not the other domains of DDX3 was indispensable to interact with ALKBH5.

### 3.3. DSBH Domain of ALKBH5 Interacted with ATP Domain of DDX3

**Figure 3:**
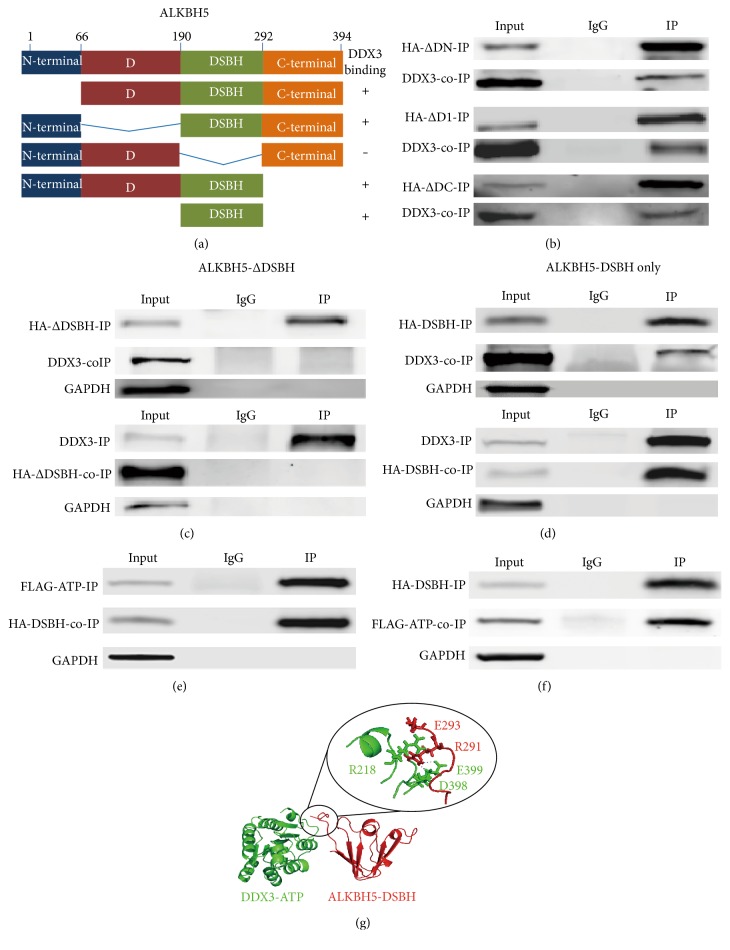
Determination of the binding domain of ALKBH5 with DDX3. (a) Schematic diagram of full-length ALKBH5 and the corresponding partial deletion constructs; summary of the results of interaction with DDX3 was shown. (b) Deletion of the N-terminal domain, D-domain, or C-terminal domain of ALKBH5 showed interaction with full-length DDX3, examined with IP and co-IP. (c) Deletion of the DSBH domain of ALKBH5 abolished its interaction with DDX3 when examined with IP and co-IP. (d) DSBH domain of ALKBH5 alone interacted with full-length DDX3, examined with IP and co-IP. (e) ATP domain of DDX3 interacted with DSBH domain of ALKBH5, examined with IP and co-IP. (f) DSBH domain of ALKBH5 interacted with ATP domain of DDX3, examined with IP and co-IP. (b–f) All IP and co-IP were performed in triplicates, and representative results are shown. (g) The predicted interaction between ATP domain of DDX3 (DDX3_211–402_) and DSBH domain of ALKBH5 (ALKBH5_190–293_). DDX3_211–402_ is labeled in green, and ALKBH5_190–293_ is labeled in red. The circle indicates the interacting region of these two domains, and a magnified view displays residues involved in formation of intermolecular hydrogen bonds. The hydrogen bonds are presented in blue dash lines.

Next, we mapped the domain in ALKBH5 responsible for its interaction with DDX3. For this purpose, 4 partially deleted constructs of ALKHB5 were constructed (Figure 3(a)). Then, each construct was examined for interaction with the full-length DDX3 using co-IP analyses. Among these constructs, deletion of the N-terminal domain, D-domain, or C-terminal domain had no effect on the binding efficiency with DDX3 (Figure 3(b)). However, when the DSBH domain of ALKBH5 was deleted, the interaction with full-length DDX3 was abolished (Figure 3(c)). Furthermore, the DSBH domain of ALKBH5 alone could interact with the full-length DDX3 (Figure 3(d)). These results showed that DSBH domain of ALKBH5 is necessary and sufficient for the interaction with DDX3. Additionally, ATP-binding domain of DDX3 and DSBH domain of ALKBH5 could interact with each other (Figures 3(e) and 3(f)).

A model illustrating interactions between DDX3_211–402_ and ALKBH5_190–293_ was predicted by Rosetta docking server (Figure 3(g)). The results imply a direct interaction between DDX3 and ALKBH5. Residues potentially essential for DDX3-ALKBH5 interactions locate in loop region of these two proteins. The model suggests hydrogen bond occurring between residue Arg218 of DDX3 and Glu293 of ALKBH5 and between Arg291 of ALKBH5 and Asp398 and Glu399 of DDX3, respectively.

### 3.4. DDX3 Modulated m^6^A RNA Demethylation

**Figure 4:**
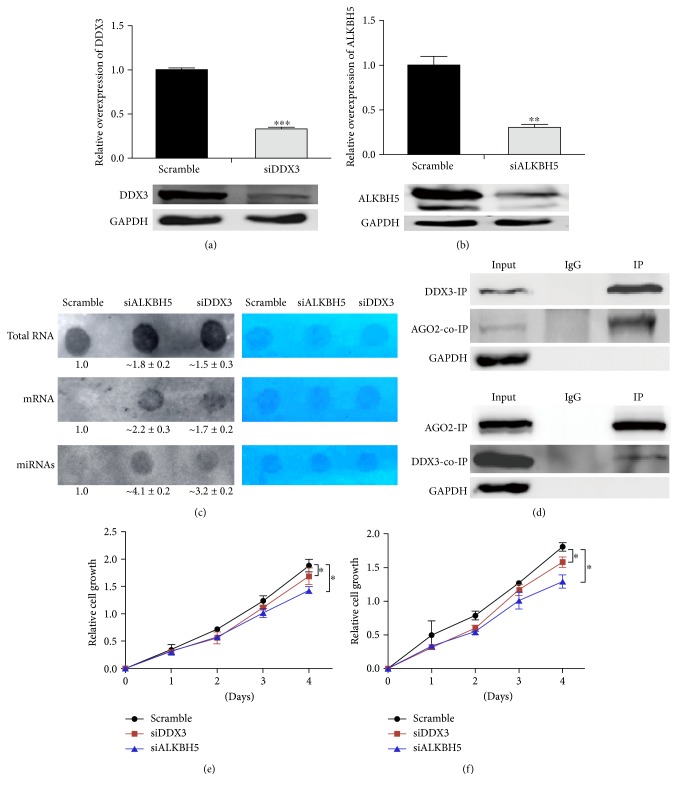
DDX3 modulated m^6^A RNA demethylation. (a) DDX3 knockdown in HEK293T cells, relative DDX3 mRNA (upper panel, qPCR), and protein levels (lower panel, Western blots) are shown. (b) ALKBH5 knockdown in HEK293T cells, ALKBH5 mRNA (upper panel, qPCR), and protein levels (lower panel, Western blots) are shown. (c) Left panel: dot-blot analyses of m^6^A levels of isolated total RNA, mRNA, and miRNA from ALKBH5 knockdown, DDX3 knockdown, and control (NC, siRNA with scrambled sequences) cells. Right panel: methylene blue staining showing equal RNA loading. (d) IP of endogenous DDX3 could co-IP endogenous AGO2, and IP of endogenous AGO2 could co-IP endogenous DDX3 in HEK293T cells. (e) Quantification of cell proliferation (MTT assay) after knockdown of DDX3 or ALKBH5 in HEK293T cells. (f) Quantification of cell proliferation (MTT assay) after knockdown of DDX3 or ALKBH5 in HeLa cells. Dot blots, IP, and co-IP were performed in triplicates, and representative results were shown. Scramble siRNA with scrambled sequences. ^∗^*P* < 0.05, ^∗∗^*P* < 0.01, and ^∗∗∗^*P* < 0.001. *P* values were determined with two-tailed Student's *t*-test. Error bars represent standard deviation (SD).

We carried out siRNA-mediated knockdown of DDX3 and ALKBH5, respectively, in HEK293T cells. The mRNA and protein levels of either DDX3 or ALKBH5 were significantly downregulated (Figures 4(a) and 4(b)). We then analyzed m^6^A modification in total RNA, mRNA, and miRNA. After knockdown of ALKBH5 or DDX3, increased m^6^A modification signals were observed (Figure 4(c)). Thus, DDX3 positively modulated the demethylation effect. It has been reported previously that DDX3 and AGO2 colocalize with each other [46] which prompted us to determine whether DDX3 could physically interact with AGO2. Indeed, in co-IP experiments, AGO2 protein was shown to interact with DDX3 (Figure 4(d)). Together, these lines of evidence suggested a possible role of DDX3 in miRNA demethylation through interacting with AGO2.

### 3.5. Knockdown of DDX3 and ALKBH5 Decreased Cell Proliferation

m^6^A RNA methylation is known to affect stem cell renewal and differentiation [34, 35]. To investigate the effect of DDX3 and ALKBH5 on cell growth, DDX3 and ALKBH5 were downregulated in HEK293T and HeLa cells with siRNAs. MTT assay showed that a significant decrease in the growth curve of both cell lines (Figures 4(e) and 4(f)). These results indicated a potential role of DDX3 and ALKBH5 in regulating cell growth, highly possibly through the modulating of m^6^A levels and in proliferating cells.

## 4. Discussion

**Figure 5:**
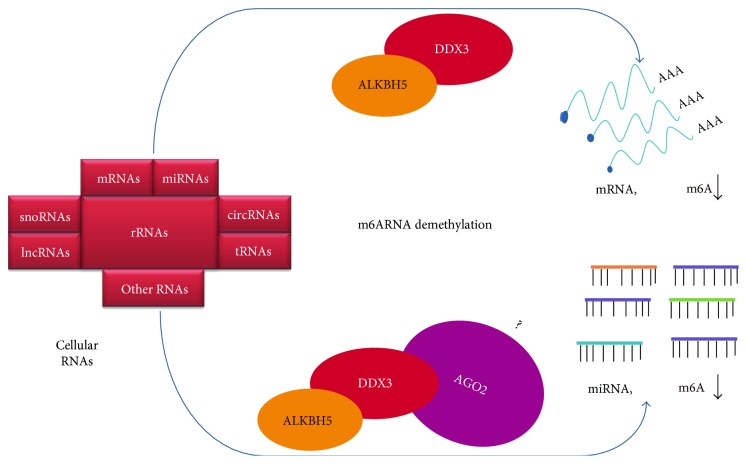
Working model for the role of ALKBH5 and DDX3 in m^6^A RNA demethylation. DDX3 interacts with ALKBH5 to regulate mRNA demethylation and additionally, by interacting with AGO2, may modulate microRNA demethylation. Whether there is direct interaction between ALKBH5 and AGO2 requires further investigations.

Our results demonstrate that DDX3 interacts with RNA demethylase ALKBH5 and AGO2. These interactions and the results of DDX3 effects on the m^6^A levels suggest a tantalizing working model that DDX3 serves as a “mediator” for the modulation of demethylation of either mRNAs or microRNAs by ALKBH5 (Figure 5).

The m^6^A is a conserved posttranscriptional modification of both coding and noncoding RNAs, which has essential roles in multiple cellular processes [20, 21, 47–49]. Recent researches have demonstrated cellular and physiological roles of m^6^A [27, 28, 50]. It is well accepted that m^6^A methylation plays critical roles in mRNA splicing and translation [27, 28]. Methylation of microRNAs has also been shown to have functional consequences [25, 26]. Various cellular conditions exhibit changes in m^6^A RNA methylation, which is associated with changes in the expression of methyltransferases and demethylases [22, 32, 34, 50]. Yet, the molecular regulatory mechanisms of either the addition or the removal of m^6^A modification require further investigations. Our results indicate that DDX3 may mediate or at least modulate the demethylation activities of ALKBH5. It seems that DDX3 is rather specific for ALKBH5 among the methyltransferases and demethylases tested (Figure 2). It is tempting to propose that other methyltransferases or demethylases may also have the corresponding specific modulators.

DDX3 plays diverse cellular functions by interacting with different proteins through its different domains. In the present study, we describe a new role for DDX3 in RNA demethylation by physical interaction with the m^6^A RNA demethylase ALKBH5 and AGO2. The ATP domain (AA 212–403) of DDX3 and the DSBH domain (AA 191–292) of ALKBH5 are responsible for their interaction (Figures 2 and 3). The conserved core segment of DDX3 (AA 227–534) is responsible for interaction with PABP1 [19]. DDX3 C-terminal region AA 260–517 fragment is required for its association with CRM1 [9]. DDX3 specifically represses cap-dependent translation by binding to eIF4E through its N-terminal 100 amino acid fragment [18]. Thus, DDX3 is involved in many biological processes via its different domains to interact with distinct proteins.

m^6^A RNA methylation is known to affect cell renewal and differentiation [31, 35]. In the present study, we have shown that knockdown of DDX3 or ALKBH5 decreases cell proliferation in both HEK293T and HeLa cell lines (Figures 4(e) and 4(f)). Interestingly, previous study has shown that BCSCs under hypoxic conditions have higher levels of ALBKH5 expression in a HIF-1*α*- and HIF-2*α*-dependent way, which eventually leads to enrichment of BCSCs in the hypoxic tumors [36]. On the other hand, previous studies have also shown that depletion of methyltransferase *Mettl13* or *Mettl14* reduced self-renewal of mouse ESCs [37].

In this study, we have identified DDX3 as a partner of ALKBH5 and AGO2 to regulate the demethylation of mRNAs and miRNAs. Further studies would help to elucidate how these interactions contribute to regulated dynamics of m^6^A epitranscriptome and the functional relevance and physiological roles of DDX3 in the context of m^6^A modification.

## 5. Conclusion

In this study, we found that DDX3 interacted with ALKBH5, an RNA m^6^A demethylase. We found ATP domain of DDX3 and DSBH domain of ALKBH5 to be indispensable to their interaction with each other. Furthermore, DDX3 could modulate the demethylation of mRNAs. We also found the interaction between DDX3 and AGO2, and DDX3 could modulate the demethylation of miRNAs. The dynamics of m^6^A RNA modification was largely elusive, and here, we showed that RNA demethylation could be regulated by proteins such as DDX3.

## Supplementary Material

Tab. S1. Oligos used in the study.
