# Supplementary material for: The DEAD-Box RNA Helicase DDX3 Interacts with m6A RNA Demethylase ALKBH5
Source: Stem Cells Int. 2017 Nov 23;2017:8596135. doi: 10.1155/2017/8596135 (PMC5733242; doi:10.1155/2017/8596135)
Supplement: Supplementary file 1 — Tab. S1. Oligos used in the study. [file 8596135.f1.pdf]

Tab. S1. Oligos used in the study.

DDX3-F	CAAGCTTGCGGCCGCGAATTCATGAGTCATGTGGCAGTGGA	Plasmid for over expression of DDX3
DDX3- R	TGGCGGCCATCGATTGAATTCTCAGTTACCCCACCAGTCAA	
ALKBH5- F	GTTCCAGATTACGCTGGATCCATGGCGGCCGCCAGCGGCTA	Plasmid for over expression of ALKBH5
ALKBH5 -R	TGGCGGCCATCGATTGAATTCTCAGTGCCGCCGCATCTTCA	
FTO- F	GTTCCAGATTACGCTGGATCCATGAAGCGCACCCCGACTGC	Plasmid for over expression of FTO
FTO- R	TGGCGGCCATCGATTGAATTCCTAGGGTTTTGCTTCCAGAA	
METTL3-F	GTTCCAGATTACGCTGGATCCATGTCGGACACGTGGAGCTC	Plasmid for over expression of METTL3
METTL3- R	TGGCGGCCATCGATTGAATTCCTATAAATTCTTAGGTTTAG	
METTL14- F	GTTCCAGATTACGCTGGATCCATGGATAGCCGCTTGCAGGA	Plasmid for Over expression of METTL14
METTL14- R	TGGCGGCCATCGATTGAATTCTTATCGAGGTGGAAAGCCAC	
WTAP- F	GTTCCAGATTACGCTGGATCCATGGGGCGGCGGTCGCGGGG	Plasmid for Over expression of WTAP
WTAP- R	TGGCGGCCATCGATTGAATTCTCACCGGGGTGGATGGACCC	
DDX3ΔN-F1	ATTCCTATTATCAAAGAGAA	DDX3 -ΔN Plasmid Construction
DDX3ΔN-R1	GAGCTATGAGAAAGCGCCACGC	
DDX3ΔN-F2	GCGTGGCGCTTTCTCATAGCTCACGCTGTAGGTATCTCAGTTC	
DDX3ΔN-R2	TTCTCTTTGATAATAGGAATCGAATTCGCGGCCGCAAGCTTG	

DDX3ΔATP-F1	TTGGCTGTAGGAAGAGTTGGC	DDX3 -ΔATP Plasmid Construction
DDX3ΔATP-R1	GGATCATGTAACTCGCCTTGATCG	
DDX3ΔATP-F2	CGATCAAGGCGAGTTACATGATCCCCCATGTTGTGCAAAAAAGC	
DDX3ΔATP-R2	GCCAACTCTTCCTACAGCCAAAGCATGCTTTTGCACTGGAGTTGG	
DDX3ΔL-F1	AACATCACACAGAAAGTAGTTTGG	DDX3 -ΔL Plasmid Construction
DDX3ΔL-R1	GGATCATGTAACTCGCCTTG	
DDX3 ΔL-F2	CAAGGCGAGTTACATGATCCCCCATGTTGTGCAAAAAAGC	
DDX3ΔL-R2	CCAAACTACTTTCTGTGTGATGTTGAAGATATATTCATCTAAGA	
DDX3ΔH-F1	TATGAACACCACTACAAGGGTAG	DDX3 -ΔH Plasmid Construction
DDX3ΔH-R1	CATTTCCGTGTCGCCCTTATTC	
DDX3-ΔH-F2	GAATAAGGGCGACACGGAAATGTTGAATACTCATACTCTTCC	
DDX3-ΔH-R2	CTACCCTTGTAGTGGTGTTCATAGTTTTCAGAGGTAGAGCCAA	
DDX3ΔC-F1	TGAGTCGACTCTAGAGGATCC	DDX3 -ΔC Plasmid Construction
DDX3ΔC-R1	GGAAATGTGCGCGGAACCCCT	
DDX3-ΔC-F2	AGGGGTTCCGCGCACATTTCCCCGAAAAGTGCCACCTGACG	
DDX3-ΔC-R2	GGATCCTCTAGAGTCGACTCAAGCCATGTTTTCTAACCAAGAC	
DDX3-ATP-F	CAAGCTTGCGGCCGCGAATTCATTCCTATTATCAAAGAGAA	DDX3 -ATP Plasmid Construction
DDX3-ATP-R	TGGCGGCCATCGATTGAATTCCAAGAAGATATATTCATCTA	
ALKBH5-ΔDN-F1	CCCGAGCGCAGCGACTATGA	ALKBH5 -ΔDN Plasmid Construction
ALKBH5-ΔDN-R2	CTCTCAAGGATCTTACCGCT	
ALKBH5-ΔDN-F2	AGCGGTAAGATCCTTGAGAGTTTTCGCCCCGAAGAACGTT	
ALKBH5-ΔDN-R2	TCATAGTCGCTGCGCTCGGGGGATCCAGCGTAATCTGGAA	

ALKBH5-ΔD1-F1	GCCGTCATCAACGACTACCAGCC	ALKBH5 -ΔD Plasmid Construction
ALKBH5-ΔD-R1	TTGCGCAACGTTGTTGCCAT	
ALKBH5-ΔD-F2	ATGGCAACAACGTTGCGCAAACTATTAACTGGCGAACTAC	
ALKBH5-ΔD-R2	GGCTGGTAGTCGTTGATGACGGCGTCCGAGTCCTCCTGATACT	
ALKBH5-ΔDSBH-F1	GAAACAAAGTCCCTGAGCAG	ALKBH5 - ΔDSBH Plasmid Construction
ALKBH5-ΔDSBH-R1	ACTTTATCCGCCTCCATCCA	
ALKBH5-ΔDSBH-F2	TGGATGGAGGCGGATAAAGTTGCAGGACCACTTCTGCGCT	
ALKBH5-ΔDSBH-R2	CTGCTCAGGGACTTTGTTTCGCTGTTGACGAAGCCCTCGG	
ALKBH5-ΔDC-F1	TGAGAATTCAATCGATGGCC	ALKBH5 - ΔDC Plasmid Construction
ALKBH5-ΔDC-R1	TCTGCTGAAGCCAGTTACCT	
ALKBH5-ΔDC-F2	AGGTAACTGGCTTCAGCAGAGCGCAGATACCAAATACTGT	
ALKBH5-ΔDC-R2	GGCGGCCATCGATTGAATTC TCACCGGGGTGCATCTAATCTTG	
ALKBH5-DSBH-F	GTTCCAGATTACGCTGGATCCGCCGTCATCAACGACTACCAGC	ALKBH5–DSBH Plasmid Construction
ALKBH5-DSBH-R	CCATCGATTGAATTCGGATCCTCACAACCGGGGTGCATCTAATC	
DDX3-F	ATGTGGCAGTGGAAAATGCG	For qPCR
DDX3-R	ATATAGCGCCCTTTGCTGGC	
ALKBH5-F	CCTGAGCAGCTCCGTGTTAC	For qPCR
ALKBH5-R	CCAGGATCCGTGGCCTGTG	
WTAP-F	GCTGGCACAGGAGGGAATA	For qPCR
WTAP-R	CCAGGTCCTTTGCTTGACTG	
METTL3-F	CCCACCTCAGTGGATCTGTT	For qPCR

METTL3-R	CCTGTGACCCAGAGGAAGAG	
FTO-F	CTGTGAAGGCCCTGAAGAGG	For qPCR
FTO-R	AAGGGGTATCGCCAAACCAG	
METTL14-F	TCCTAATTCCTACTTGACTGGTTGT	For qPCR
METTL14-R	CCTCCACGGCCCCCTCTA	
GAPDH-F	TCGTCCCGTAGACAAAATGGT	For qPCR
GAPDH-R	CGCCCAATACGGCCAAA	
siDDX3	CAAAGGGCGCUAUAUUCCUCCUCAU<br>AUGAGGAGGAAUAUAGCGCCCUUUG	siRNA of DDX3
siALKBH5	CCCAUCGUGUCCGUGUCCUUCUUUA<br>UAAAGAAGGACACGGACACGAUGGG	siRNA of ALKBH5
scrambled	UUCUCCGAACGUGUCACGUTT<br>ACGUGACACGUUCGGAGAATT	Negative control
